# RhoA/rock signaling mediates peroxynitrite-induced functional impairment of Rat coronary vessels

**DOI:** 10.1186/s12872-016-0372-6

**Published:** 2016-10-11

**Authors:** Zhijun Sun, Xing Wu, Weiping Li, Hui Peng, Xuhua Shen, Lu Ma, Huirong Liu, Hongwei Li

**Affiliations:** 1 Department of Heart Center, Capital Medical University Affiliated Beijing Friendship Hospital, Beijing, China; 2 Beijing Key Laboratory of Metabolic Disturbance Related Cardiovascular Disease, Beijing, People’s Republic of China

**Keywords:** Peroxynitrite, RhoA/ROCK, Hyperglycemia, Coronary artery, Vasoconstriction, Vasodilation

## Abstract

**Background:**

Diabetes-induced vascular dysfunction may arise from reduced nitric oxide (NO) availability, following interaction with superoxide to form peroxynitrite. Peroxynitrite can induce formation of 3-nitrotyrosine-modified proteins. RhoA/ROCK signaling is also involved in diabetes-induced vascular dysfunction. The study aimed to investigate possible links between Rho/ROCK signaling, hyperglycemia, and peroxynitrite in small coronary arteries.

**Methods:**

Rat small coronary arteries were exposed to normal (NG; 5.5 mM) or high (HG; 23 mM) D-glucose. Vascular ring constriction to 3 mM 4-aminopyridine and dilation to 1 μM forskolin were measured. Protein expression (immunohistochemistry and western blot), mRNA expression (real-time PCR), and protein activity (luminescence-based G-LISA and kinase activity spectroscopy assays) of RhoA, ROCK1, and ROCK2 were determined.

**Results:**

Vascular ring constriction and dilation were smaller in the HG group than in the NG group (*P* < 0.05); inhibition of RhoA or ROCK partially reversed the effects of HG. Peroxynitrite impaired vascular ring constriction/dilation; this was partially reversed by inhibition of RhoA or ROCK. Protein and mRNA expressions of RhoA, ROCK1, and ROCK2 were higher under HG than NG (*P* < 0.05). This HG-induced upregulation was attenuated by inhibition of RhoA or ROCK (*P* < 0.05). HG increased RhoA, ROCK1, and ROCK2 activity (*P* < 0.05). Peroxynitrite also enhanced RhoA, ROCK1, and ROCK2 activity; these actions were partially inhibited by 100 μM urate (peroxynitrite scavenger). Exogenous peroxynitrite had no effect on the expression of the voltage-dependent K^+^ channels 1.2 and 1.5.

**Conclusions:**

Peroxynitrite-induced coronary vascular dysfunction may be mediated, at least in part, through increased expressions and activities of RhoA, ROCK1, and ROCK2.

## Background

Diabetes mellitus (DM) is associated with disturbances in coronary arterial function that contribute to the detrimental effects of DM on the heart. Indeed, DM has been reported to cause dysfunction of endothelial-dependent vasodilation of small coronary arteries [1–3], even during the early stages of the disease [4]. This impairment in the relaxation of coronary vessels may involve, at least in part, reduced availability of nitric oxide (NO) [5, 6]. In turn, this is thought to be due to a decrease in NO synthase (NOS) activity [7, 8], as well as to the interaction of NO with superoxide to generate peroxynitrite (ONOO^−^) [9]. Peroxynitrite is a powerful oxidizing agent that causes nitration of aromatic amino acid residues, forming 3-nitrotyrosine (3-NT)-modified proteins. Enhanced 3-NT formation has been reported in an animal model of DM [10]. Peroxynitrite is known to participate in DM-induced endothelial dysfunction [11, 12]. Indeed, studies in DM models showed that increases in peroxynitrite levels were associated with vascular permeability and impaired vasorelaxation [13]. It has also been shown that peroxynitrite suppresses eNOS expression through RhoA activation, leading to endothelial dysfunction [11]. Peroxynitrite leads to the formation of nitrotyrosine in the artery walls, which is directly toxic to endothelial cells and leads to endothelial dysfunction [12]. The presence of nitrotyrosine is associated with microvascular anomalies in DM, and correlate with blood glucose [14].

RhoA is a small, guanosine-5’-triphosphate binding protein, bound to the plasma membrane. Upon stimulation, RhoA activates RhoA-associated protein kinase (ROCK), of which there are two isoforms (ROCK1 and ROCK2) with an overall homology of 65 % [15]. RhoA/ROCK signaling regulates numerous cellular processes, including gene transcription, organization of the actin cytoskeleton, and cell contraction, adhesion, motility, proliferation, and differentiation [16]. The RhoA/ROCK signaling pathway has been involved in several pathological conditions [16, 17] including hypertension [18, 19], atherosclerosis [20], stroke [21], coronary vasospasm [22], angina [23], ischemia-reperfusion injury [24], and heart failure [25, 26]. There is strong evidence that DM increases the expressions and activities of RhoA and ROCK in various tissues, and that the resulting phosphorylation of downstream targets enhances the contraction of vascular smooth muscle cells [27–33].

Potassium channels regulate K^+^ efflux and are major regulators of membrane potential of vascular smooth muscle cells. Therefore, K^+^ channel activity is an important factor involved in the regulation of vasoconstriction and blood vessel diameter [34]. Voltage-dependent K^+^ channels (K_v_) limit membrane depolarization to maintain the vascular tone [35]. K_v_1.2 and K_v_1.5 are subunits that are expressed in vascular smooth muscle cells [35]. Many vasoconstrictors act through the inhibition of K^+^ channel activity [35].

However, the precise mechanisms by which peroxynitrite impairs the function of small coronary arteries remain largely unknown. The aim of the present study was to explore whether the RhoA/ROCK signaling pathway and K_V_ are involved in mediating peroxynitrite-induced impairment of rat small coronary arteries.

## Methods

### Animals

Male Sprague–Dawley rats (age, 7–8 weeks; weight, 180–220 g) were provided by Vital River Laboratory Animal Technology Co., Ltd. (Beijing, China). Animals were housed under specific pathogen-free conditions and given free access to food and water. All animal experiments were performed in accordance with the Guidelines for the Care and Use of Laboratory Animals, Ministry of Science and Technology (Beijing, China). The study was approved by the Animal Care and Use Committee of Beijing Friendship Hospital, Capital Medical University (Beijing, China).

### Preparation of isolated rat small coronary arteries

Rats were anesthetized with chloral hydrate (0.5 mL/100 g i.p.) and received heparin (2500 U/kg) to inhibit blood clot formation. The heart was removed and placed in HEPES-buffered (Hanks) solution at 4 °C. The cardiac apex and ascending aorta were fixed using pins, and the left auricle was identified under a 2 × 10 stereoscopic microscope (with a halogen lamp as a cold light source; Nikon, Tokyo, Japan). The left anterior descending branch of the coronary artery was identified under the left auricle, and small coronary arteries with diameters ≤200 μm were isolated rapidly as vascular rings of about 2-mm. The endothelium was denuded with air, and denudation was verified by failure to dilate in response to 1 μM acetylcholine.

For the experiments, the isolated vascular rings were incubated in 6-well dishes at 37 °C for 24 h with Dulbecco’s modified Eagle’s medium (DMEM; Gibco, USA) supplemented with 20 % fetal calf serum (Gibco, USA), 100 U/mL penicillin G, and 100 mg/mL streptomycin. The vascular rings were divided into the following groups: normal glucose (NG group, 5.5 mM D-glucose), L-glucose (LG group, 5.5 mM D-glucose plus 17.5 mM L-glucose), or high glucose (HG group, 23 mM D-glucose). In experiments of pharmacologic disruption of the RhoA/ROCK pathway, vascular rings of the HG group were pre-treated for 16 h with either the RhoA inhibitor, C3 transferase (1 μg/ml, HG + C3 group; Cytoskeleton, USA), or the ROCK inhibitor, Y-27632 (10 μM, HG + Y-27632 group; Sigma-Aldrich, St. Louis, MO, USA), followed by culture in high glucose medium. In experiments investigating the effects of exogenous peroxynitrite, vascular rings of the NG group were incubated (37 °C, 24 h) in the presence of additional agents: 5 μM peroxynitrite (ONOO^−^ group), 5 μM decomposed peroxynitrite (DC-ONOO^−^ group), or 5 μM peroxynitrite plus 100 μM urate (ONOO^−^ + urate group). Peroxynitrite was synthesized according to published methods, and determined spectrophotometrically using the reported extinction coefficient for peroxynitrite (1670/M/cm) [36]. Before each application, the stock solution was diluted in 1 mM NaOH and rapidly added to the chamber to achieve a final concentration of 5 μM. Decomposed peroxynitrite was made by leaving peroxynitrite at room temperature for at least 2 h. Urate (Sigma-Aldrich) was used as a scavenger of peroxynitrite.

### Contraction and relaxation of vascular rings in response to 4-aminopyridine and forskolin

After incubation with the appropriate experimental solution, each coronary artery ring was threaded onto two tungsten filaments (each with a diameter of 40 μm) and fixed to the bath transducers of a Multi Wire Myograph System-610 M (DMT, Aarhus, Denmark). The vascular ring was bathed in HEPES-buffered solution (8.415 g/l NaCl, 0.432 g/l KCl, 0.244 g/l MgCl_2_ · 6H_2_O, 0.277 g/l CaCl_2_, 2 g/l glucose, 1.1915 g/l HEPES) gassed with 100 % O_2_ and maintained at 37 °C. As a standardization procedure, the transmural pressure of the vascular ring was set to a baseline value of 13.33 kPa (100 mmHg); this was achieved by adjusting the tension of the vascular ring to the desired value, with the aid of the following equations:

$$ {\mathrm{P}}_{\mathrm{i}}=2\uppi {\mathrm{T}}_{\mathrm{i}}/\mathrm{I}{\mathrm{C}}_{\mathrm{i}} $$

$$ {\mathrm{T}}_{\mathrm{i}}={\mathrm{F}}_{\mathrm{i}}/2\mathrm{L} $$

$$ \mathrm{I}{\mathrm{C}}_{\mathrm{i}}=205.6+2{\mathrm{X}}_{\mathrm{i}} $$

where:

P_i_ (kPa) = effective transmural pressure

T_i_ (mN/mm) = vascular ring tension per unit length

IC_i_ (μm) = vascular ring inner perimeter

F_i_ (mN) = total tension of the vascular ring, determined by the myography system

X_i_ (μm) = distance between the two tungsten wires, determined by the myography system

L (mm) = vascular ring length, determined with a dissecting microscope and micrometer

Experiments were performed after an equilibration period of 60–90 min, during which the bathing medium was replaced every 15–20 min with pre-heated (37 °C) HEPES-buffered solution. The tension changes of each vascular ring were determined automatically by the myography system and recorded by a computer running the Chart 5.5 software (ADInstruments, Dunedin, New Zealand). Before examining the responses of the vascular rings to vasoactive substances, the rings were exposed repeatedly to 120 mM KCl until the contraction amplitude differences for three successive applications of KCl were less than 10 %; tension was then allowed to stabilize for 30 min before vasoactive substances were administered. Contraction was measured in response to a range of concentrations (0.1–3 mM) of 4-aminopyridine (4-AP, Sigma-Aldrich), a blocker of the K_v_1 K^+^ channels. Relaxation in response to 1 μM forskolin (an adenylyl cyclase activator; Sigma-Aldrich) was also assessed.

### Isolation of vascular smooth muscle cells from rat small coronary arteries

Small coronary arteries with diameters ≤200 μm were obtained as described above. Coronary vascular smooth muscle cells (VSMCs) were isolated enzymatically. The adventitia was dissected from the small coronary arteries, and the vessel washed three times with sterile Hanks’ buffered salt solution. The vessel was incubated for 10 min at room temperature with 1 mL of phosphate-buffered saline (PBS) containing 0.1 % bovine serum albumin (BSA), and subsequently digested for 10 min at 37 °C in 1 mL of PBS containing 0.15 % papain, 0.1 % dithioerythritol (DTE), and 0.1 % BSA. The vessel was then incubated for 10 min at 37 °C with 1 mL of PBS containing 0.2 % collagenase, 0.05 % elastase, and 0.1 % soybean trypsin inhibitor. Subsequently, 5 mL of DMEM containing 20 % fetal bovine serum (FBS) were added to stop digestion. The mixture was centrifuged at 1000 rpm for 7 min, and the supernatant was discarded. The pellet was washed with sterile PBS and resuspended in 4–5 mL of culture medium. The resuspended cells were seeded into culture flasks and cultured for 24 h (DMEM containing 20 % FBS). When the cells were fully adherent, as determined under an inverted microscope, the culture medium was changed to DMEM containing 10 % FBS, 100 U/mL of penicillin G, and 100 μg/mL of streptomycin. Primary VSMCs were cultured for 7 days to reach a logarithmic phase. The cell passages were performed when cell coverage reached 80 %. Culture medium was discarded, and the cells were washed with sterile PBS and digested with 1 mL of 0.25 % trypsin (2–3 min, 37 °C). Digestion was terminated with culture medium containing FBS when the morphology of 80–90 % of the cells changed from spindle-shaped to round. The digested cells were seeded into flasks for culture, and passages could be performed after 3–4 days. Cells were then used for glucose exposure experiments.

Smooth muscle cells were identified by immunofluorescence. Single-cell suspensions were seeded into dishes containing coverslips, and a coverslip covered with a monolayer of cells was fixed with 95 % pre-cooled ethanol for 30 min. After three washes with PBS, the cells were incubated with the primary antibody (mouse anti-rat alpha-smooth muscle actin polyclonal antibody; Santa Cruz Biotechnology, Santa Cruz, CA, USA) for 30 min at 37 °C. After three washes with PBS, the cells were incubated with the secondary antibody (IgG-FITC-labeled chicken anti-mouse secondary antibody; Santa Cruz Biotechnology) for 30 min at 37 °C. After washing with PBS (three times), 4′,6-diamidino-2-phenylindole (DAPI) was added for 2 min (to stain the nuclei). After three washes with PBS, the coverslip was mounted onto a slide with mounting medium, and the cells were observed using a fluorescence microscope (Nikon, Tokyo, Japan).

### Real-time PCR for mRNA expression

Total RNA of cultured coronary vascular smooth muscle cells was extracted with Trizol (Invitrogen, Carlsbad, CA, USA), and RNA concentration and purity were determined using an ultraviolet spectrophotometer. Total RNA (1 μg) was reverse-transcribed into cDNA with reverse transcriptase (Shanghai Jierdun Biotech Co. Ltd., Shanghai, China), according to the manufacturer’s protocol. The expressions of RhoA, ROCK1, ROCK2, K_v_1.2, and K_v_1.5 mRNA were assessed by real-time polymerase chain reaction (PCR) of the cDNA (2 μg), using an ABI Step One Plus Real-Time-PCR System (Applied Biosystems, Foster City, CA, USA), SYBR Green Master Mix (Applied Biosystems), and the following primers (Invitrogen):

RhoA (103 bp):

- F: 5′-CATCCCAGAAAAGTGGACTCCA- 3′
  R: 5′-CCTTGTGTGCTCATCATTCCG- 3′

ROCK1 (113 bp):

- F: 5′-GAATGACATGCAAGCGCAAT- 3′
  R: 5′-GTCCAAAAGTTTTGCACGCA- 3′

ROCK2 (150 bp):

- F: 5′-GAAACAACTGGATGAAGCTAATGC- 3′
  R: 5′-GTTTCAAGCAGGCAGTTTTTATCTT- 3′

K_v_1.2:

- F: 5′CGT CAG CTT CTG TCT GGA AAC C 3′
  R: 5′TGC ATG TCC TCG TTC TCA TCC 3′

K_v_1.5:

- F: 5′-CCTGTCCCCGTCATCGTCTC- 3′
  R: 5′-ACCTTCCGTTGACCCCCTGT- 3′

GAPDH (75 bp):

- F: 5′-CCTGCCAAGTATGATGACA- 3′
  R: 5′- GTAGCCCAGGATGCCC - 3′

GAPDH was used as a reference to obtain the relative fold changes for the target genes, using the comparative Ct method. Relative mRNA expressions were estimated using the 2^-△△CT^ method.

### Immunohistochemistry

Standard immunohistochemistry protocols were applied to small coronary arteries treated for 24 h with the various experimental solutions, in order to determine the protein expression levels of RhoA, ROCK1, and ROCK2. The small coronary arteries were fixed with 4 % paraformaldehyde and sectioned (4 μm). The following primary antibodies (100 μL) were used: anti-RhoA (1:200 dilution; BS1782; Bioworld Technology Inc., St. Louis Park, MN, USA), anti-ROCK1 (1:200 dilution; sc-6055; Santa Cruz Biotechnology), and anti-ROCK2 (1:250 dilution; sc-1851; Santa Cruz Biotechnology). The secondary antibody was biotinylated goat anti-rabbit IgG (Beyotime Institute of Biotechnology, Shanghai, China). Sections were visualized with diaminobenzidine (DAB; Shanghai Jierdun Biotech) and counterstained with hematoxylin and eosin (H&E). Images were captured with a digital camera (Nikon) and analyzed using the IMS imaging processing system (Shanghai Jierdun Biotech). Positively stained regions were counted and analyzed. Cardiomyocytes were excluded.

### Immunoprecipitation

Adherent cells were cultured in 10-cm dishes for protein isolation. The cell culture medium was discarded. The cells were washed with PBS, and 1 mL of lysis buffer was added for cell lysis. A 200–1000 μL sample containing about 200–1000 μg of protein was mixed with 1 μg of IgG (that shared the same host species as the IgG used in the immunoprecipitation) and 20 μL of protein A/G agarose. The mixture was incubated at 4 °C for 30–120 min, with gentle agitation, and then centrifuged at 1000 *g* for 5 min. The supernatant was isolated for subsequent protein immunoprecipitation. Primary antibodies (0.2–2 μg) against RhoA (1:400), ROCK1 (1:400), ROCK2 (1:400), K_v_1.2 (1:500), or K_v_1.5 (1:500) were added to the supernatant, and the mixture was incubated overnight at 4 °C with gentle agitation. Then, 20 μL of thoroughly resuspended protein A/G agarose was added, and this was incubated for a further 1–3 h at 4 °C, with gentle agitation. The mixture was centrifuged at 1000 *g* for 5 min or transiently at high speed, and the supernatant was discarded. The pellet was washed five times with 0.5–1 mL of lysis buffer (used for protein isolation) or PBS, and resuspended in 20–40 μL of 1× SDS-PAGE loading buffer. After transient centrifugation at high speed, the sample was boiled at 100 °C for 3–5 min. Partial or total samples were used for protein quantification or SDS-PAGE electrophoresis, or stored at −20 °C for later use.

### Western blotting for protein expression

Total protein was extracted from artery rings, and the protein concentration determined using a bicinchoninic acid (BCA) protein assay kit (Thermo Fisher Scientific, Waltham, MA, USA). Immunoblotting was performed with the following primary antibodies: anti-RhoA (Bioworld), anti-ROCK1 (Santa Cruz Biotechnology), anti-ROCK2 (Santa Cruz Biotechnology), anti- K_v_1.2, or anti-K_v_1.5. HRP-conjugated anti-rabbit secondary antibody was used at a dilution of 1:2000. Detection of GAPDH (diluted 1:1500; #5471; Cell Signaling Technology, Danvers, MA, USA) served as an internal loading control. All blots were scanned with the LabWorks image processing system (UVP Inc., Upland, CA, USA). Protein band pixel values were calculated using Gel-pro Analyzer 4.0 (Media Cybernetics Inc., Rockville, MD, USA).

### Measurement of RhoA and ROCK activities

RhoA activation was measured using the luminescence-based G-LISA Activation Assay kit (Cytoskeleton Inc., Denver, CO, USA), according to the manufacturer’s instructions. Values were normalized to the protein content using a colorimetric assay (Bio-Rad Laboratories, Hercules, CA, USA), according to the manufacturer’s recommendations. ROCK1 and ROCK2 activities were detected using the Kinase Activity Spectroscopy Kit (GMS50184.3 for ROCK1 and GMS50184.1 for ROCK2; Genmed Scientific Inc., Arlington, MA, USA), according to the manufacturer’s instructions.

### Statistical analysis

Continuous data were expressed as means ± standard deviation (SD). Statistical analyses were performed using SPSS 18.0 (IBM, Armonk, NY, USA). Comparisons between groups were performed using one-way analysis of variance (ANOVA) or the Student’s *t*-test, as appropriate. The Bonferroni correction was applied when comparing three or more groups. *P* values <0.05 were considered statistically significant.

## Results

### Impairment of vascular ring contraction and dilation by high glucose or peroxynitrite may involve RhoA/ROCK signaling

**Fig. 1:**
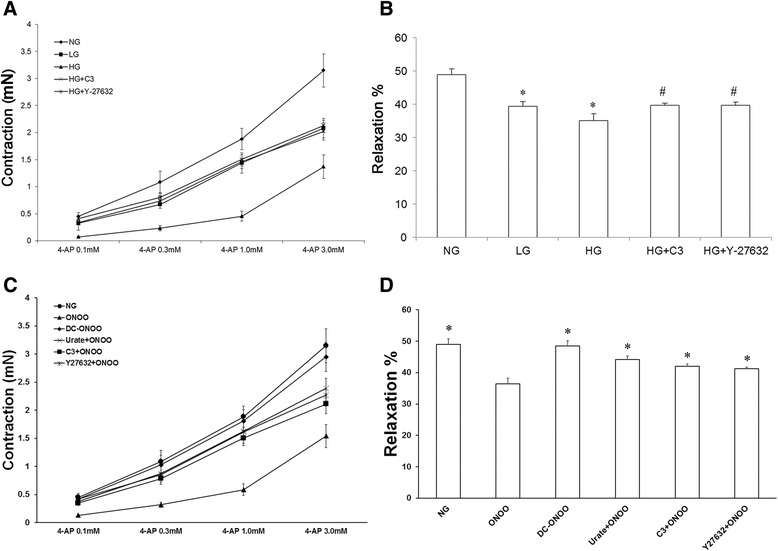
Impairment of vascular ring contraction and dilation by high glucose involves RhoA/ROCK signaling. **a** Concentration-response curves showing the contractile responses of rat small coronary artery vascular rings treated with 4-AP (a voltage-gated K^+^ channel blocker; 0.1–3 mM) under various experimental conditions. Treatment with high glucose (23 mM D-glucose) was associated with attenuation of contraction of rings treated with 4-AP (compared with 5.5 mM D-glucose) that was partially reversed by C3 transferase (a RhoA inhibitor) and Y-27632 (a ROCK inhibitor). **b** Rat small coronary artery vascular rings treated with high glucose (23 mM D-glucose) showed an impairment of dilation (compared with 5.5 mM D-glucose) in response to 1 μM forskolin (adenylyl cyclase activator); this impairment was partially reversed by C3 transferase and Y-27632. Data are shown as mean ± SD (*n* = 6). *P < 0.05 vs. NG. ^#^ *P* < 0.05 vs. HG. **c** Concentration-response curves showing the contractile responses of rat small coronary artery vascular rings to 4-AP (a voltage-gated K^+^ channel blocker; 0.1-3 mM) under various experimental conditions. Treatment with peroxynitrite (5 μM peroxynitrite) was associated with attenuation of contraction of rings treated with 4-AP (compared with 5.5 mM D-glucose and 5 μM decomposed peroxynitrite) that was partially reversed by urate, C3 transferase, and Y-27632. **d** Rat coronary small coronary artery rings treated with ONOO (5 μM peroxynitrite) showed an impairment of dilation (compared with 5.5 mM D-glucose and 5 μM decomposed peroxynitrite) in response to 1 μM forskolin (adenylyl cyclase activator); this impairment was partially reversed by urate, C3 transferase, and Y-27632. Data are shown as mean ± SD (*n* = 6). *P < 0.05 vs. ONOO

Figure 1a presents concentration-response curves showing the contractile responses of vascular rings in the various experimental groups treated with 4-AP. Maximal contraction in response to 3 mM 4-AP, was significantly smaller in the LG (2.08 ± 0.17 mN) and HG (1.37 ± 0.22 mN) groups than in the NG group (3.15 ± 0.31 mN) (*P* < 0.05; *n* = 6). Interestingly, inhibition of RhoA or ROCK partially reversed this effect of high glucose: the maximal contractile response of rings treated with 4-AP was significantly larger in the HG + C3 (2.13 ± 0.09 mN) and HG + Y-27632 (2.02 ± 0.16 mN) groups than in the HG group (*P* < 0.05; *n* = 6). Similar observations were made in experiments using forskolin (Fig. 1b): dilation in response to 1 μM forskolin was significantly reduced in the LG (39.47 ± 1.32 %) and HG (35.20 ± 1.98 %) groups compared with the NG group (48.97 ± 1.77 %), and was higher in both the HG + C3 (39.80 ± 1.59 %) and HG + Y-27632 (39.68 ± 1.57 %) groups than in the HG group (*P* < 0.05; *n* = 6).

Additional experiments were carried out to determine whether exogenous peroxynitrite (5 μM) could mimic these effects of high glucose. The contractile response of rings treated with 3 mM 4-AP was significantly reduced in the ONOO^−^ group (1.54 ± 0.21 mN) compared with the NG group (3.15 ± 0.31 mN) (*P* < 0.05; *n* = 6), but DC-ONOO^−^ was without effect (2.95 ± 0.26 mN). Furthermore, the attenuation of contraction by ONOO- was inhibited (albeit not completely) by urate (2.39 ± 0.18 mN), C3 transferase (2.17 ± 0.15 mN), and Y-27632 (2.27 ± 0.10 mN) (P < 0.05 compared with the ONOO^−^ group; *n* = 6) (Fig. 1c). Similarly, relaxation to forskolin was significantly smaller in the ONOO^−^ group (36.37 ± 1.80 %) than in the NG (48.97 ± 1.77 %), DC-ONOO^−^ (48.55 ± 1.64 %), ONOO^−^ + urate (44.17 ± 1.14 %), ONOO^−^ + C3 (42.02 ± 1.73 %), and ONOO^−^ + Y-27632 (41.22 ± 1.53 %) groups (P < 0.05; n = 6) (Fig. 1d).

### Treatment with high glucose increases protein expressions of RhoA, ROCK1, and ROCK2 detected by immunohistochemistry in denuded vessels

**Fig. 2:**
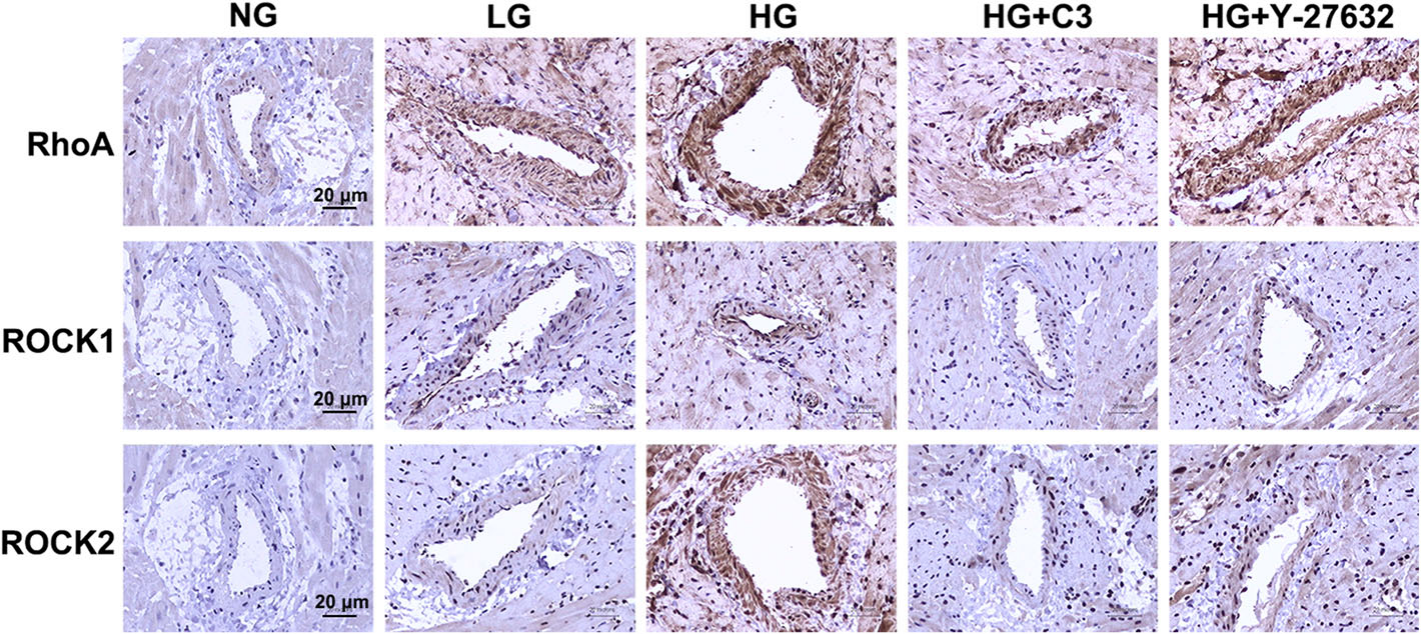
Immunohistochemistry showing protein expressions of RhoA, ROCK1, and ROCK2 in rat coronary small coronary artery rings. Sections were stained with rabbit primary antibodies against RhoA, ROCK1, or ROCK2, followed by goat anti-rabbit biotinylated secondary antibody, and then visualized with diaminobenzidine. Sections were counterstained with hematoxylin and eosin. Brown staining in the image is indicative of expression of the protein of interest. Treatment with high glucose (23 mM D-glucose) was associated with enhanced expressions of RhoA, ROCK1, and ROCK2 compared with normal glucose (5.5 mM). These effects of high glucose were partially reversed by C3 transferase (a RhoA inhibitor) and Y-27632 (a ROCK inhibitor). NG: 5.5 mM glucose; HG: 23 mM D-glucose; HG + C3: 23 mM D-glucose and 1 μg/ml C3 transferase; HG + Y27632: 23 mM D-glucose and 10 μM Y-27632. Magnification: ×200

**Fig. 3:**
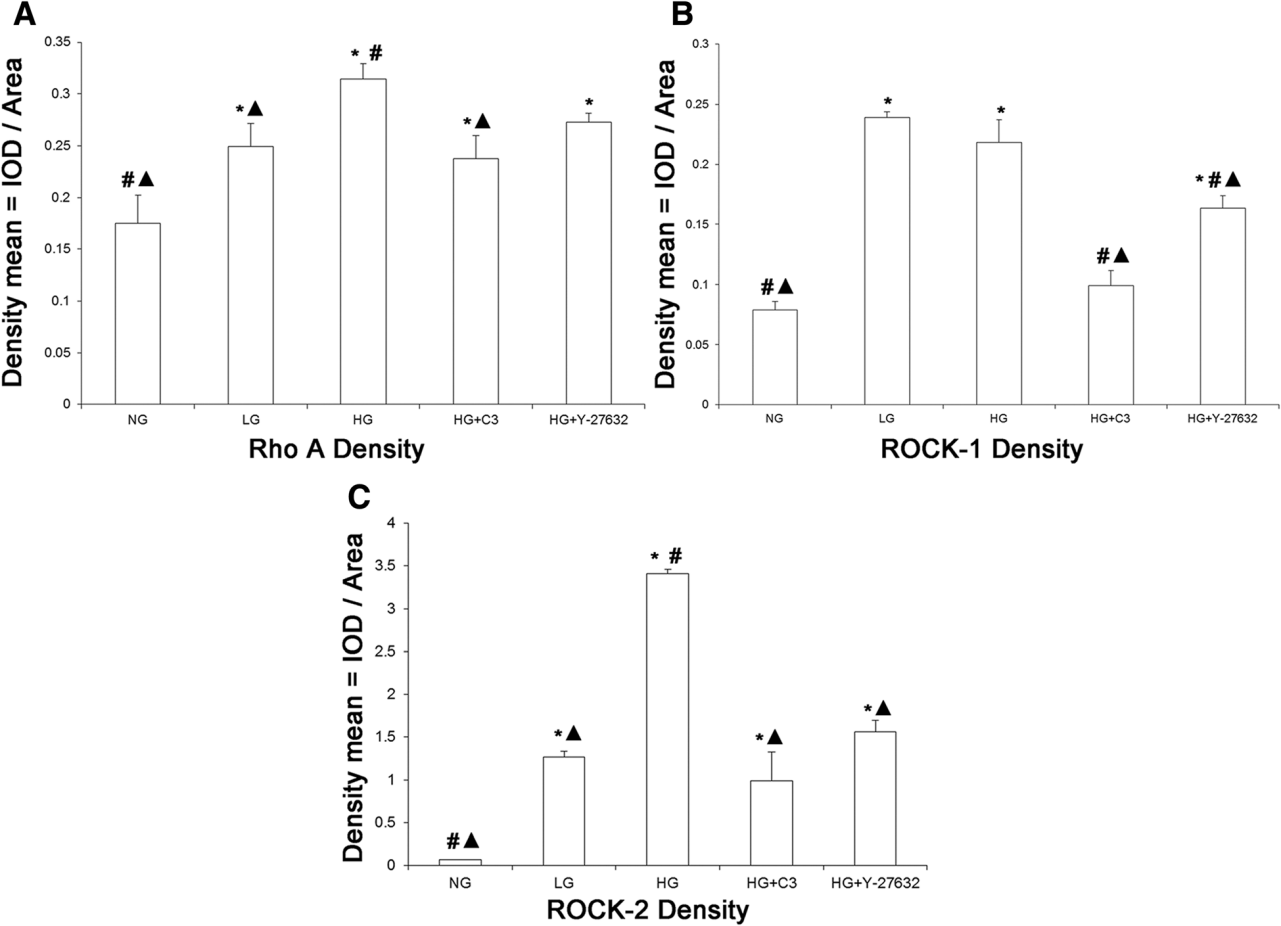
Mean protein expressions of RhoA, ROCK1, and ROCK2 in rat small coronary artery vascular rings determined from immunohistochemistry experiments. **a** Protein expression of RhoA. **b** Protein expression of ROCK1. **c** Protein expression of ROCK2. NG: 5.5 mM glucose; LG: 5.5 mM D-glucose plus 17.5 mM L-glucose; HG: 23 mM D-glucose; HG + C3: 23 mM D-glucose and 1 μg/ml C3 transferase; HG + Y27632: 23 mM D-glucose and 10 μM Y-27632. Data are shown as mean ± SD (*n* = 6 rats, 5–6 vessels from each). * *P* < 0.05 compared with the NG group; ^#^ *P* < 0.05 compared with the LG group; ^▲^ *P* < 0.05 compared with the HG group. Cardiomyocytes were excluded

Figure 2 shows representative images obtained from immunohistochemistry experiments carried out to detect the protein expressions of RhoA, ROCK1, and ROCK2 in the various groups. Quantitative analyses of the immunohistochemistry data are shown in Fig. 3. Protein expressions of RhoA, ROCK1, and ROCK2 were significantly higher in the HG group than in the NG group (*P* < 0.05; *n* = 3). Importantly, inhibition of RhoA (C3 transferase) or ROCK (Y-27632) significantly attenuated the upregulation of ROCK1 and ROCK2 induced by high glucose, while Y-27632 inhibited the upregulation of RhoA (*P* < 0.05; *n* = 3). This suggests that an increase in RhoA/ROCK signaling induced by high glucose can in turn feedback to upregulate the protein expressions of RhoA, ROCK1, and ROCK2.

### Treatment with high glucose increases mRNA and protein expressions of RhoA, ROCK1, and ROCK2 detected by real-time PCR and Western blotting

**Fig. 4:**
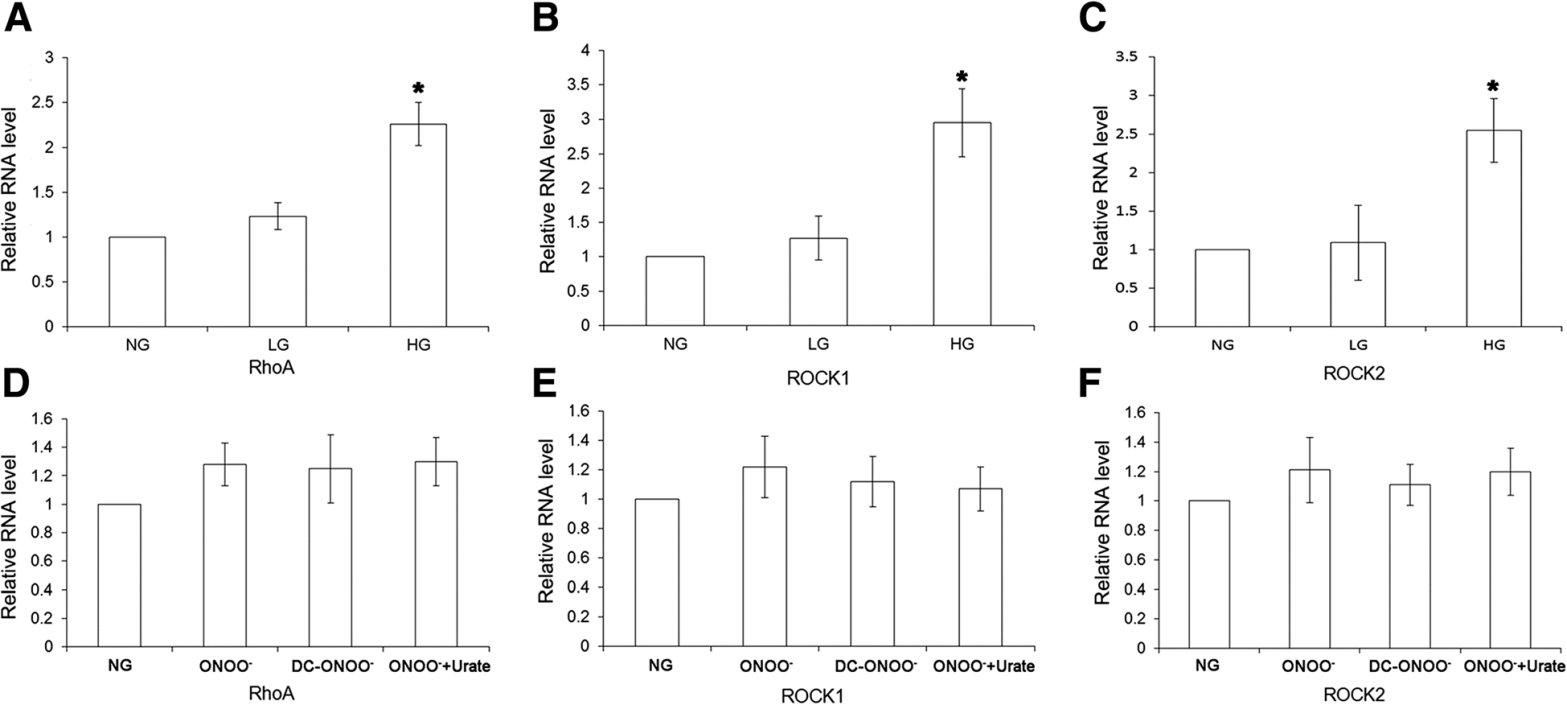
mRNA expressions of RhoA, ROCK1, and ROCK2 in rat coronary vascular smooth muscle cells. mRNA expression was determined using real-time PCR. Treatment with high glucose (23 mM D-glucose) resulted in an increase in the mRNA expression of RhoA (**a**), ROCK1 (**b**), and ROCK2 (**c**). However, exogenous peroxynitrite was without significant effect on the mRNA expression of RhoA (**d**), ROCK1 (**e**), and ROCK2 (**f**). NG: 5.5 mM glucose; LG: 5.5 mM D-glucose plus 17.5 mM L-glucose; HG: 23 mM D-glucose; ONOO: 5 μM peroxynitrite; DC-ONOO: 5 μM decomposed peroxynitrite; ONOO + Urate: 5 μM peroxynitrite and 100 μM urate (to scavenge peroxynitrite). Data are shown as mean ± SD (*n* = 5). * *P* < 0.05 compared with the NG group

**Fig. 5:**
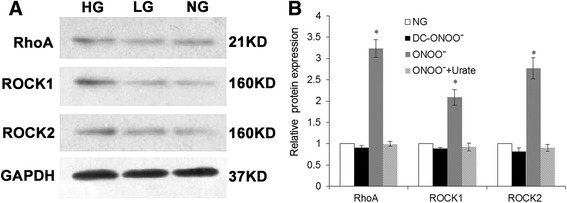
Protein expressions of RhoA, ROCK1 and ROCK2 in rat coronary vascular smooth muscle cells. Protein expression was determined using the Western blot technique. **a** Representative blots for RhoA, ROCK1, and ROCK2; GAPDH expression was used as an internal reference. **b** Mean data for protein expression levels. Treatment with peroxynitrite was associated with enhanced the expression of RhoA, ROCK1, and ROCK2 compared with controls. NG: 5.5 mM glucose; LG: 5.5 mM D-glucose plus 17.5 mM L-glucose; HG: 23 mM D-glucose. Data are shown as mean ± SD (*n* = 5). * *P* < 0.05 compared with the NG group

The mRNA expression of RhoA, ROCK1, and ROCK2, detected using real-time PCR, was significantly enhanced by high glucose (*P* < 0.05; *n* = 5; Fig. 4a–c). No significant effects of exogenous peroxynitrite were observed (Fig. 4d–f), although a trend toward a small increase in expression could not be excluded. Consistent with the immunohistochemistry data, high glucose significantly enhanced the protein expressions of RhoA, ROCK1, and ROCK2 measured using western blotting (*P* < 0.05; *n* = 5; Fig. 5).

### Treatment with high glucose or exogenous peroxynitrite increases the activity of RhoA, ROCK1, and ROCK2

**Fig. 6:**
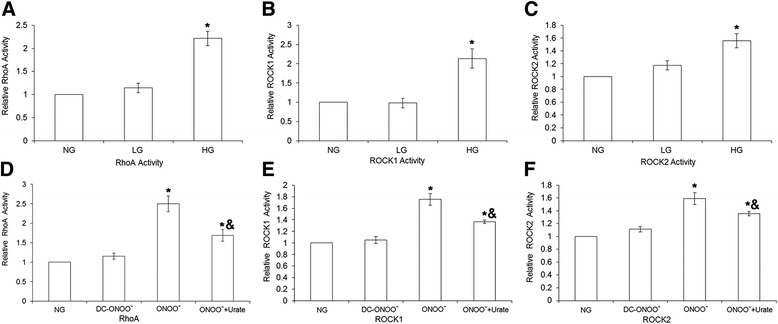
RhoA, ROCK1, and ROCK2 activities in rat coronary vascular smooth muscle cells. RhoA activation was measured using the luminescence-based G-LISA Activation Assay kit; ROCK1 and ROCK2 activities were detected using the Genmed Kinase Activity Spectroscopy Kit. Treatment with high glucose (23 mM D-glucose) resulted in an increase in the activities of RhoA (**a**), ROCK1 (**b**), and ROCK2 (**c**). Similarly, treatment with 5 μM peroxynitrite was associated with increases in RhoA (**a**), ROCK1 (**b**), and ROCK2 (**c**) activities; these effects could be partially inhibited by 100 μM urate (a scavenger of peroxynitrite). NG: 5.5 mM glucose; LG: 5.5 mM D-glucose plus 17.5 mM L-glucose; HG: 23 mM D-glucose; ONOO^−^: 5 μM peroxynitrite; DC-ONOO: 5 μM decomposed peroxynitrite; ONOO + Urate: 5 μM peroxynitrite and 100 μM urate (to scavenge peroxynitrite). Data are shown as mean ± SD (*n* = 5). * *P* < 0.05 compared with the NG group; ^&^ *P* < 0.05 compared with the ONOO^−^ group

Further studies were undertaken to determine whether the high glucose-induced upregulation of RhoA, ROCK1, and ROCK2 expression translated into enhanced activities. As shown in Fig. 6a–c, treatment with high glucose was associated with significant increases in RhoA, ROCK1, and ROCK2 activity (*P* < 0.05; *n* = 5). Interestingly, 5 μM peroxynitrite (but not decomposed peroxynitrite) mimicked the effects of high glucose, and these actions of exogenous peroxynitrite were partially inhibited by urate (Fig. 6d–f).

### mRNA and protein expressions of K_v_1.2 and K_v_1.5 are not affected by exogenous peroxynitrite

**Fig. 7:**
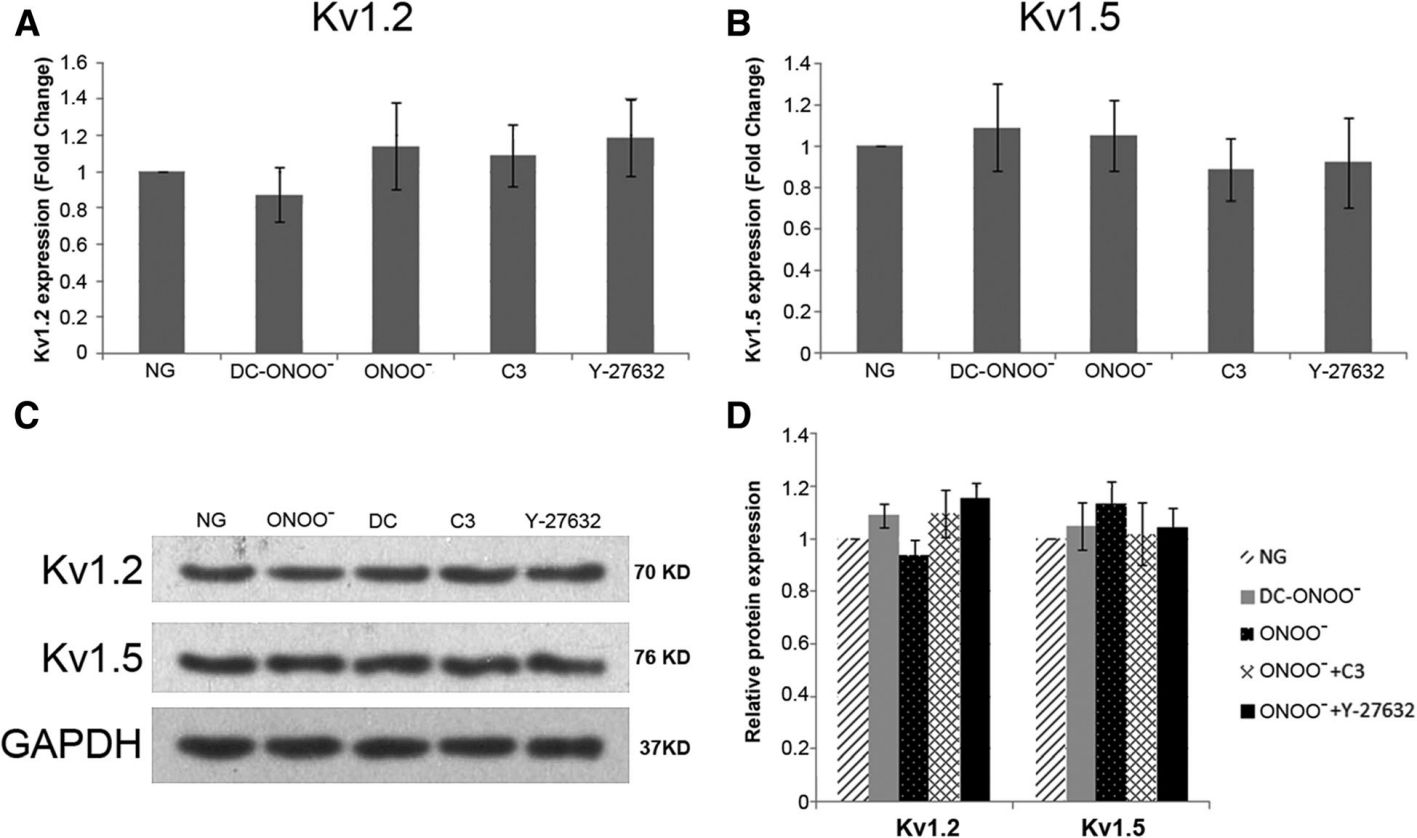
mRNA and protein expression of K_v_1.2 and K_v_1.5 in coronary vascular smooth muscle cells. **a** K_v_1.2 mRNA expression (real-time PCR) was not affected by 5 μM peroxynitrite. **b** K_v_1.5 mRNA expression (real-time PCR) was not influenced by 5 μM peroxynitrite. **c** Representative blots for K_v_1.2 and K_v_1.5. GAPDH expression was used as an internal reference. **d** Mean data for protein expression levels. Peroxynitrite (5 μM) had no effect on the protein expression of K_v_1.2 and K_v_1.5. NG: 5.5 mM glucose; ONOO^−^: 5 μM peroxynitrite; DC-ONOO: 5 μM decomposed peroxynitrite; C3 or ONOO^−^ + C3: 5 μM peroxynitrite and C3 transferase (to inhibit RhoA); Y-27632 or ONOO^−^ + Y-27632: 5 μM peroxynitrite and Y-27632 (to inhibit ROCK). Data are shown as mean ± SD (*n* = 5)

To investigate whether the functional impairment of vascular ring contraction/relaxation by high glucose might involve peroxynitrite-mediated changes in the expressions of K_v_, the mRNA and protein expression of K_v_1.2 and K_v_1.5 were determined. As shown in Fig. 7, exogenous peroxynitrite (5 μM) had no significant effects on the mRNA and protein expression of K_v_1.2 and K_v_1.5 (*n* = 5).

### Exogenous peroxynitrite induces 3-NT-modification of proteins

**Fig. 8:**
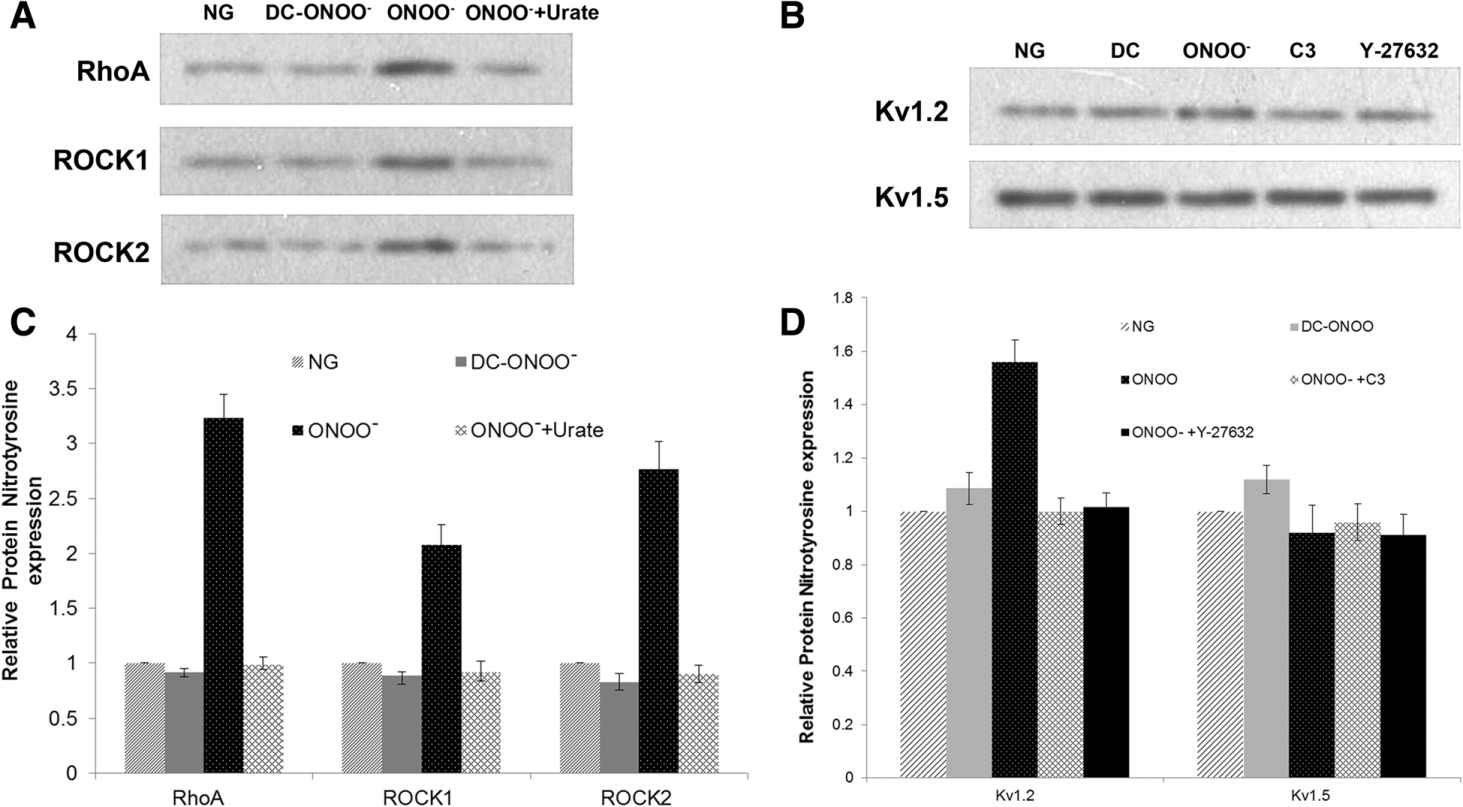
Nitrotyrosine-modification of proteins by peroxynitrite. 3-NT-modified proteins were detected by immunoprecipitation. **a** Representative blots showing detection of 3-NT-modified RhoA, ROCK1, and ROCK2. **b** Representative blots showing detection of 3-NT-modified K_v_1.2 and K_v_1.5. **c** Quantification of 3-NT-modified RhoA, ROCK1, and ROCK2. 5 μM peroxynitrite (but not decomposed peroxynitrite) induced an increase in the levels of 3-NT-modified RhoA, ROCK1, and ROCK2; this was reversed by 100 μM urate (a scavenger of peroxynitrite). **d** Quantification of 3-NT-modified K_v_1.2 and K_v_1.5. 5 μM peroxynitrite (but not decomposed peroxynitrite) induced an increase in the levels of 3-NT-modified K_v_1.2; this was reversed by 100 μM urate. In contrast, peroxynitrite had no effect on levels of 3-NT-modified K_v_1.5. NG: 5.5 mM glucose; ONOO^−^: 5 μM peroxynitrite; DC-ONOO: 5 μM decomposed peroxynitrite; ONOO + Urate: 5 μM peroxynitrite and 100 μM urate (to scavenge peroxynitrite); C3 or ONOO^−^ + C3: 5 μM peroxynitrite and C3 transferase (to inhibit RhoA); Y-27632 or ONOO^−^ + Y-27632: 5 μM peroxynitrite and Y-27632 (to inhibit ROCK). Data are shown as mean ± SD (*n* = 5). * *P* < 0.05 compared with NG; ^&^ *P* < 0.05 compared with ONOO^−^

Exogenous peroxynitrite (5 μM) caused significant increases in the levels of 3-NT-modified RhoA, ROCK1, ROCK2, and K_v_1.2 (*P* < 0.05; *n* = 5; Fig. 8). However, no effect on K_v_1.5 was observed (Fig. 8).

## Discussion

The aim of the present study was to explore whether the RhoA/ROCK signaling pathway is involved in mediating peroxynitrite-induced impairment of rat small coronary arteries. The main findings were that short-term exposure to high glucose and peroxynitrite caused functional impairment of rat small coronary artery rings that was partially reversed by RhoA or ROCK inhibition, and enhancement of RhoA, ROCK1, and ROCK2 activity. Therefore, increased RhoA, ROCK1, and ROCK2 expression and/or activities may contribute to peroxynitrite-induced coronary vascular dysfunction. Although the underlying mechanisms remain to be established, the effects of peroxynitrite may involve 3-NT modification of RhoA, ROCK1, ROCK2, and/or other proteins, which in turn enhances RhoA/ROCK signaling. Vascular ring increase in constrictor tone to 4-AP treatment was smaller in HG and peroxynitrite-treated vessels, meaning that Kv channels contribute less to the maintenance of dilator tone in HG or peroxynitrite incubated vessels than in NG vessels.

The interaction of NO with superoxide reduces NO availability (which contributes to coronary vascular dysfunction) and generates peroxynitrite that causes nitration of various proteins [5, 6, 9]. Increased 3-NT formation has been observed in an animal model of DM [10], and peroxynitrite has been suggested to target subcellular compartments in vascular endothelium [37]. Interestingly, the enzymes involved in NO synthesis are compartmentalized in caveolae [38], and the impairment of flow-mediated dilation of small coronary arteries in patients with DM may be due to disruption of caveolae by peroxynitrite and hence endothelial NOS uncoupling [39]. Our observations that peroxynitrite mimicked the effects of high glucose (in terms of coronary arteriolar dysfunction) suggest that nitration of cellular proteins contributes to the detrimental effects of DM on the coronary circulation, but these findings must be taken with caution because there was no endothelial contribution in the present study.

RhoA/ROCK signaling is involved in numerous cellular processes, and may play a role in several pathological conditions [16, 17]. Various studies have reported that DM increases the expression and activity of RhoA and ROCK [27–33], consistent with the effects of HG observed in the present study. Both ROCK1 and ROCK2 are expressed in vascular endothelial and smooth muscle cells [33, 40]. In models of DM, ROCK inhibition improves microvascular damage, enhances cerebral vasodilation, reverses vasoconstriction, and improves coronary dysfunction through an enhancement of endothelial NOS [26, 27, 32, 33, 41, 42]. An impairment of endothelial NOS by RhoA and ROCK inhibitors would mitigate against peroxynitrite-induced disruption of caveolae and endothelial NOS uncoupling [39], potentially underlying our observations that C3 transferase and Y-27632 attenuated peroxynitrite-induced vascular dysfunction. But again, the effect of endothelium could not be assessed in the present study, and additional observations are necessary.

The findings that impaired vascular function and increased RhoA, ROCK1, and ROCK2 expression/activity occurred after only a 24-h exposure to HG imply that activation of the RhoA/ROCK pathway is an early event in the pathogenesis of diabetic complications. Endothelial-dependent vasodilation is compromised during the early stages of DM [11, 43]. Interestingly, in a rat model of early-stage DM, there was a trend toward upregulation of ROCK1 and ROCK2 expression, and a larger effect of ROCK inhibition (with fasudil) in the presence of NOS and cyclooxygenase blockade; furthermore, fasudil inhibited the occurrence of focal and segmental constrictions [44]. The involvement of RhoA/ROCK signaling in the early stages of DM would make ROCK a promising therapeutic target for treating diabetic complications [16, 45].

Hyperglycemia has been suggested to stimulate RhoA/ROCK signaling through the generation of reactive oxygen species (ROS) [46]. Interestingly, peroxynitrite may be a mediator of enhanced ROCK signaling in endothelial cells [40, 47], in agreement with the present findings. Although the mechanisms remain unclear, peroxynitrite and the Rho/ROCK pathway may interact via a positive feedback loop, as has been proposed for the relationship between Rho/ROCK, protein kinase C-β_2_ (PKCβ_2_), inducible NOS (iNOS), and ROS. PKCβ_2_ [48] is believed to contribute to diabetic complications, and inhibitors of this kinase can improve DM-induced retinopathy, cardiomyopathy, nephropathy, and neuropathy [49, 50]. Hyperglycemia is thought to activate PKCβ_2_, which in turn activates iNOS and RhoA/ROCK signaling [51–53]. PKCβ_2_ inhibition reduces iNOS-mediated cardiovascular abnormalities in diabetic rats [52]. iNOS may contribute to enhanced RhoA and ROCK expressions/activities; consistent with our observations, ROCK inhibition can downregulate RhoA expression [53]. Rats with experimental DM show increases in cardiac ROCK2 expression and PKCβ_2_ expression and activity; intriguingly, ROCK2 appears to directly interact with and activate PKCβ_2_ through phosphorylation at the T641 site [51]. Thus, RhoA/ROCK may contribute to cardiac dysfunction in DM by activating PKCβ_2_ and generating ROS, through a positive-feedback loop involving iNOS [54]. ROS may contribute to the PKCβ_2_ activation [55] and iNOS upregulation [56], while the effects of PKCβ_2_ may, in part, be mediated by ROS [57, 58] and induction of iNOS [52]. Further research is required to establish whether peroxynitrite-induced activation of the RhoA/ROCK pathway leads to alterations in NO signaling (such as iNOS induction) that exacerbate the generation of peroxynitrite. In addition, other signaling mechanisms may be involved in alterations to NO signaling, such as the JNK and TGFβ/SMAD pathways [59].

K_V_ are involved in the regulation of vascular tone by limit membrane depolarization [35]. A recent study has shown that diabetes led to reduced presence of K_V_1.2 in nerves from diabetic mice and diabetic patients [60]. Another study suggests that diabetes might be associated with gender-specific decreases in K_V_1.5 levels in myocytes from male mice, and that this effect might be triggered by the renin-angiotensin system [61]. In the present study, exogenous peroxynitrite had no significant effects on the mRNA and protein expression of K_v_1.2 and K_v_1.5, but significantly increased the levels of 3-NT-modified K_v_1.2. A previous study revealed that excess peroxynitrite production might impair K_V_ function in DM, which is consistent with the present study [62]. Another study has shown that peroxynitrite over-production induced by high glucose impaired K_V_-mediated vascular dilation mediated by cAMP [63]. 4-AP is a K_V_ blocker that allow the separation of the effects of K_V_ and Ca^2+^-activated K^+^ channels [35]. In the present study, 4-AP decreased the contraction response to a greater extent in glucose-treated rings compared with controls. Forskolin is an activator of adenylyl cyclase, leading to an increase in intracellular cAMP levels. The decreased vasorelaxation observed in the present study also point toward a role of K_V_ in the process of impaired vascular response in DM. Taken together, these results suggest that K_v_ channels contribute less to the maintenance of dilator tone in HG or peroxynitrite incubated vessels than in NG vessels. However, further study is necessary to adequately assess the role and exact mechanisms of K_V_ in DM and vascular dysfunction.

## Conclusions

Vascular ring increase in constrictor tone to 4-AP treatment was smaller in HG and peroxynitrite-treated vessels, meaning that Kv channels contribute less to the maintenance of dilator tone in HG or peroxynitrite incubated vessels than in NG vessels, possibly via 3-NT modification of RhoA, ROCK1, ROCK2, KV1.2, and/or other proteins. Importantly, these results suggest that even a short-term exposure to glucose was might be sufficient to induce this impaired response.

## Abbreviations

ANOVA: Analysis of variance
BSA: Bovine serum albumin
DAB: Diaminobenzidine
DM: Diabetes mellitus
DMEM: Dulbecco’s modified Eagle’s medium
FBS: Fetal bovine serum
H&E: Hematoxylin and eosin
HG: High glucose
iNOS: Inducible NOS
LG: L-glucose
NG: Normal glucose
NO: Nitric oxide
NOS: NO synthase
PCR: Polymerase chain reaction
PKCβ2: Protein kinase C-β2
ROCK: RhoA-associated protein kinase
ROS: Reactive oxygen species
SD: Standard deviation
VSMCs: Vascular smooth muscle cells
